# Altered processing of consecutive changeable emotional voices in individuals with autistic traits: behavioral and ERP studies

**DOI:** 10.1186/s40359-025-02452-2

**Published:** 2025-03-17

**Authors:** Chao Huo, Chunyan Meng, Huiling Qian, Wanchen Li, Min Shao, Yujuan Huang, Jing Meng

**Affiliations:** 1 Research Center for Brain and Cognitive Science, Chongqing Normal University, Chongqing, 401331 China; 2 Key Laboratory of Applied Psychology, Chongqing Normal University, Chongqing, 401331 China; 3 School of Psychology, Shenzhen University, Shenzhen, 518060 China; 4 Nanchong Vocational College of Science and Technology, Nanchong, 637200 China; 5 School of Educational Sciences, Chongqing Normal University, Chongqing, 401331 China; 6 Guizhou Light Industry Technical College, Guiyang, 550025 China

**Keywords:** Autism, Autistic traits, Emotion, Auditory, ERP

## Abstract

**Background:**

Similar to individuals with autism spectrum disorder (ASD), individuals with autistic traits are expected to exhibit alterations in emotion recognition. However, many previous studies using single emotional stimuli did not observe these alterations in such individuals. Given that consecutive changeable emotional stimuli are more common in social interactions than single emotional stimuli, impaired mental processing of consecutive changeable emotions may be a key factor underlying the social interaction challenges faced by these individuals.

**Methods:**

The present research aimed to investigate the behavioral and neural responses to consecutive changeable emotional voices in individuals with autistic traits through two studies (Study 1 and Study 2). Based on the autism-spectrum quotient (AQ) scores, participants were categorized into two groups: the High-AQ and the Low-AQ groups. In Study 1, both groups were asked to judge a single emotional voice (positive, negative, or neutral; S1) presented in each trial in Task 1, or the last presented emotional voice (S3) in a triplet of stimuli (S1-S2-S3, trains of three consecutive changeable emotional voices) in Task 2. In Study 2, both groups were instructed to passively listen to the stimulus triplet (S1-S2-S3), and event-related potential (ERP) technology was used to investigate their neural responses to each stimulus.

**Results:**

No significant group difference was found in response to S1 voices in either Study 1 or Study 2. However, the High-AQ group elicited higher arousal levels (Study 1) and larger P2 amplitudes (Study 2) in response to S3 emotional voices (positive and negative) compared to the Low-AQ group.

**Conclusion:**

These findings reveal that individuals with autistic traits may exhibit alterations in their processing of consecutive changeable emotions in the auditory modality.

## Introduction

Autism spectrum disorder (ASD) is a neurodevelopmental condition with a polygenic predisposition, characterized by pervasive difficulties in reciprocal social behaviors and communication, as well as repetitive or stereotyped behaviors [1]. Previous research has established that autistic traits exist on a continuum in both typically developing individuals and individuals with ASD, spanning a spectrum of severity from mild to severe [2, 3]. The autism-spectrum quotient (AQ) [4] is frequently utilized to quantify autistic traits. Recent studies have shown that individuals with ASD typically exhibit AQ scores at the higher end of the distribution [4, 5], while individuals with autistic traits in the typically developing population generally have AQ scores that are close to, but below, the clinical threshold for ASD diagnosis [4, 6]. Previous studies have documented that individuals with ASD and individuals with autistic traits share similar genetic and etiological factors [7, 8], behavioral patterns [9], personality traits [10], and phenotypic heterogeneity [11], suggesting that investigating autistic traits in the general population may provide valuable insights into understanding the etiology and nature of ASD [12, 13].

To our knowledge, understanding and processing others’ emotions are crucial for social communication and interaction [14–16], thus difficulties in emotion recognition and processing are considered core deficits in ASD [17]. Previous studies have investigated emotion recognition in individuals with ASD and autistic traits using static and single emotional stimuli in laboratory settings within the visual modality [18–21], and found no significant difficulties in recognizing most individual emotions [22–27]. However, using single emotional stimuli in the visual modality during experiments may overlook potential critical cues for emotion recognition in real social contexts [28]. In everyday life, people frequently encounter a series of continuous, dynamic, and complex emotional stimuli, which require relatively more mental resources to process than single emotional stimuli [29], thereby adding complexity to real-world social interactions. Consequently, some researchers have employed stimuli or methods with higher ecological validity, making them more representative of everyday life [30], and found that individuals with ASD and autistic traits exhibit altered emotional processing and recognition in the visual modality [31–33]. For instance, when presented with dynamic stimuli or videos containing sequences of emotional information in the visual modality, these individuals face significant challenges in processing these emotions [33–36]. Therefore, these individuals may exhibit altered processing patterns when handling consecutive changeable emotions in the visual modality.

Importantly, emotional cues in daily life are not limited to the visual modality alone; auditory signals, especially human voices in the auditory modality, play a complementary and critical role in conveying emotions. Voices provide both verbal and nonverbal information about the speaker’s emotional state, identity, and intent [37], and are essential for social interaction and emotional communication [38]. Unlike visual cues, auditory cues may trigger stronger emotional reactions in individuals with ASD, who often exhibit heightened sensitivity and lower tolerance to aversive voices in daily life, leading to observable distress and avoidance behaviors [39, 40]. Additionally, individuals with ASD or autistic traits show no significant differences compared to control groups in reaction time and accuracy when processing simple, single emotional voices, but exhibit altered neural habituation and difficulties in recognizing intense emotional voices, especially when these voices are complex or presented in repeated or consecutive sequences [41, 42]. These findings suggest that the auditory modality may pose unique challenges to emotional processing in individuals with ASD or autistic traits. Therefore, studying how these individuals process consecutive changeable emotional cues in the auditory modality is crucial for a comprehensive understanding of emotional processing in ASD.

Our previously published ERP study has thoroughly investigated the behavioral and neural responses of individuals with autistic traits to consecutive changeable emotional stimuli in the visual modality [23]. In this study, we employed the S1-S2-S3 paradigm, adapted from previously published ERP studies in auditory [41] and sensory modalities [43, 44], in which each recorded section presented triplets of identical or different stimuli (S1-S2-S3). Previous studies primarily used triplets of identical stimuli to investigate neural habituation in response to repeated stimuli [41], while triplets of different stimuli were employed to examine whether physical properties (e.g., intensity, interstimulus intervals) of stimulus sequences could influence neural habituation [43, 44]. To avoid neural habituation effects [43, 44] and better simulate sequences of consecutive changeable emotional cues encountered by individuals with autistic traits in daily social interactions [29], which often pose significant processing challenges [33–36], we presented participants with pseudo-randomized, sequential trains of three facial expressions (positive, neutral, negative) from the same individual [23]. The findings of this study showed no significant difference between participants with high AQ scores (High-AQ group) and low AQ scores (Low-AQ group) to S1 faces (first presented stimuli). However, the High-AQ group displayed larger P1 amplitudes to S3 faces (third presented stimuli) and larger P3 amplitudes to negative S3 faces than the Low-AQ group. P1 amplitude is thought to reflect the early automatic processing of faces, and is modulated by the low-level visual features of faces [45, 46]. P3 amplitude reflects the late-stage emotional encoding of emotional processing [47] and is associated with arousal levels [48, 49]. Thus, these results suggest that individuals with autistic traits may exhibit neural responses to single emotional stimuli in the visual modality similar to those of individuals with low AQ scores, but show heightened arousal levels in response to consecutive changeable emotional faces. However, whether these effects can be detected in the auditory modality remains unknown.

Thus, the current research with two studies (Study 1 and Study 2) was carried out to investigate how individuals with autistic traits process consecutive changeable emotions in the auditory modality. In Study 1, a behavioral study consisting of two tasks was designed to explore the behavioral responses of individuals with autistic traits to single emotional voices (Task 1) and consecutive changeable emotional voices (Task 2). Building on the results of Study 1, Study 2 further explored the neural mechanisms underlying the processing of consecutive changeable emotional voices in individuals with autistic traits. Additionally, as individuals with autistic traits have been found to exhibit altered top-down attention when instructed by experimental tasks [41, 50], Study 2 employed a passive listening task to avoid interference from explicit instructions and explore authentic neural responses under naturalistic conditions [41]. Emotional voices have been shown to elicit the frontal-central N1, P2, and the late negative component (LNC), reflecting physical properties [51, 52], arousal levels [53–55], and late stages of emotional processing [56], respectively. Based on previous findings, we predicted that individuals with autistic traits would exhibit heightened behavioral and neural responses to consecutive changeable emotional voices, especially in terms of arousal levels, but their behavioral and neural responses to single emotional voices would be similar to those of individuals with low AQ scores.

## Study 1: behavioral study

### Method

#### Participants

The Mandarin version [57] of the AQ questionnaire [4] was administered to assess the autistic traits of 1,167 typically developing university students at Chongqing Normal University in China. According to previous studies [20, 41, 50], participants were randomly selected from the top 10% and the bottom 10% of AQ scores to form the High-AQ and Low-AQ groups, respectively. A priori power analysis, conducted using G*Power 3, determined that a sample size of 28 participants was necessary to achieve a statistical power of 0.95. This was to detect median-sized effects (f = 0.25) with an alpha value of 0.05 for a three-way ANOVA involving two within-participant factors and one between-participant factor. To mitigate the potential impact of dropouts or errors during the study, Study 1 enrolled 58 participants (High-AQ group: *n* = 29, 14 females; Low-AQ group: *n* = 29, 14 females), aged 18–25 years (*Mean* = 19.81 years, *SD* = 1.26 years). Inclusion criteria encompassed normal hearing and no history of medical, neurological, or psychiatric conditions. No participants were previously diagnosed with ASD. Further details can be found in Table 1.

**Table 1:** Ages and AQ scores of the High-AQ and Low-AQ groups in study 1

Group	Age (years)						AQ score				
	Min	Max	*Mean* ± *SD*	*t*	*p*		Min	Max	*Mean* ± *SD*	*t*	*p*
High-AQ	18	22	19.79 ± 0.98	-0.10	0.918		29	35	30.86 ± 1.77	37.33	< 0.001
Low-AQ	18	25	19.83 ± 1.51				8	15	12.93 ± 1.89		

Note: AQ, Autism Spectrum Quotient. The statistics results (*p*-values and *t*-values) were conducted using independent samples *t*-tests between High-AQ and Low-AQ groups

Following the principles of the Declaration of Helsinki, all participants were provided with detailed information about the experimental procedures before the commencement of the study. Subsequently, they provided their informed consent by signing a consent form. Ethical approval for the study was obtained from the Chongqing Normal University Research Ethics Committee. All procedures were conducted in strict accordance with ethical guidelines and regulations.

#### Stimuli

A total of 24 audio recordings of interjections (/α/) with three different emotional voices, namely positive/happy (8 recordings), neutral (8 recordings), and negative/sad voices (8 recordings), were selected from the Montreal Affective Voices database [58]. These recordings were performed by eight actors (four males and four females). To maintain consistency with prior research [21, 59], all audio recordings were edited to a duration of 1000 ms, with a mean intensity of 70 dB. Before the experiment, all audio recordings were evaluated by 40 undergraduate students (20 females) who did not participate in the main experiment. They were asked to rate these emotional voices on 9-point Likert scales for valence (1 = *very unhappy*, 5 = *neutral*, 9 = *very happy*) and arousal (1 = *extremely peaceful*, 9 = *extremely excited*). The results showed significant differences in emotional valence ratings among the three types of emotional voices (*F*_*2*, 38_ = 64.83, *p* < 0.001, η^*2*^_p_ = 0.77). Post hoc tests revealed that positive voices (6.84 ± 1.22) were more positive than neutral voices (4.77 ± 0.31, *p* < 0.001) and negative voices (2.92 ± 1.09, *p* < 0.001), and neutral voices were more positive than negative voices (*p* < 0.001). A significant difference in arousal ratings was also found across emotional types (*F*_*2*, 38_ = 39.72, *p* < 0.001, η^*2*^_p_ = 0.68). Post hoc tests indicated that arousal ratings were higher for both positive (6.06 ± 1.63, *p* < 0.001) and negative voices (6.11 ± 1.52, *p* < 0.001) compared to neutral voices (4.46 ± 1.48), whereas no significant difference was found between positive and negative voices (*p* = 1).

#### Experimental procedure

**Fig. 1:**
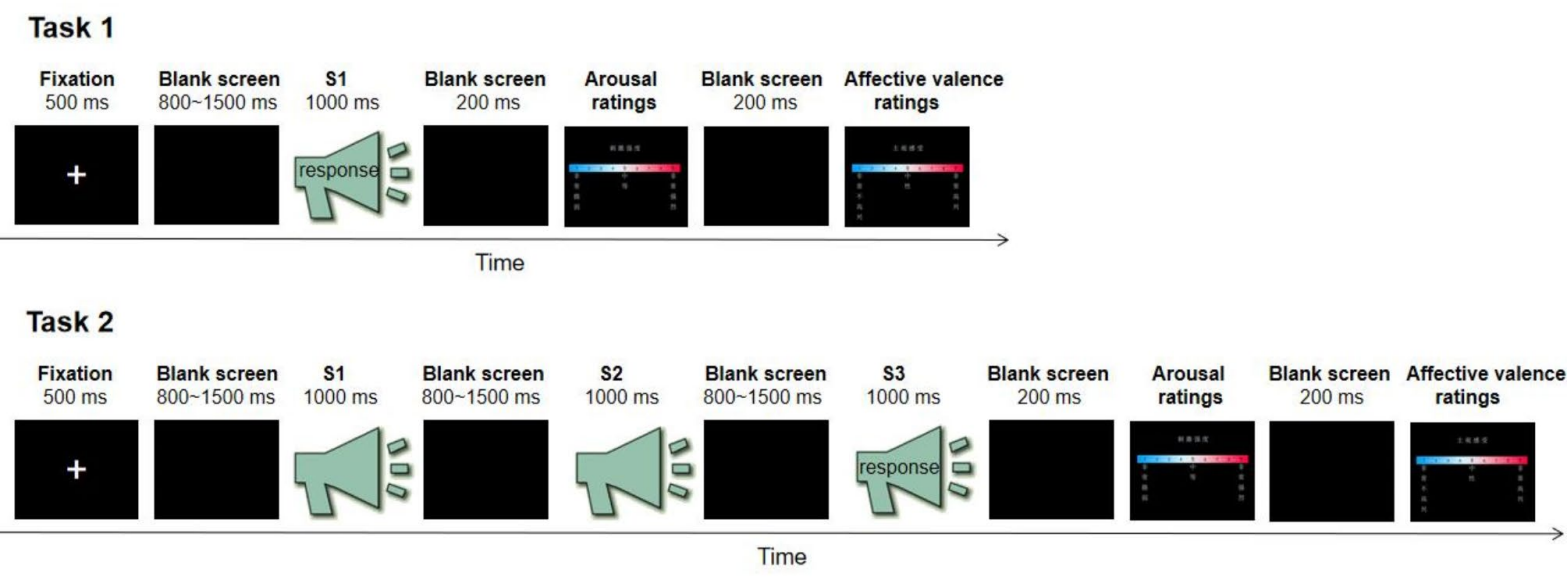
Experimental flowchart for study 1

The experiment took place in a quiet, soundproof room with a comfortable ambient temperature. Stimuli were presented in a pseudo-random order using the E-Prime 3.0 program. Participants engaged in two experimental tasks: Task 1 and Task 2 (see Fig. 1). The two tasks were presented in a block design, with one task completed prior to proceeding to the other. The order of these tasks was counterbalanced across participants. Before the experiment, each participant underwent a training session to familiarize themselves with the procedures.

According to previous studies [50, 60], an example trial of Task 1 is displayed in the top panel of Fig. 1. At the beginning of each trial, a fixation cross appeared on the screen for 500 ms. Following this, a blank screen was presented for a duration ranging from 800 ms to 1500 ms. Subsequently, an emotional voice (S1) was presented for 1000 ms. Participants were instructed to judge as accurately and quickly as possible whether the voice was positive, neutral, or negative by pressing a key (“1”, “2”, or “3”). Key-pressing was counterbalanced across participants to control for potential order effects. Once participants pressed a key or if no response was made within 3 s after the voice presentation, a 200 ms blank screen appeared. Following this, participants were instructed to rate the arousal (1 = *extremely peaceful*, 9 = *extremely excited*) and affective valence (1 = *very unhappy*, 5 = *neutral*, 9 = *very happy*) on the keyboard after listening to the voice. The inter-trial interval was randomly varied from 1000 ms to 1500 ms. Task 1 comprised a total of 48 trials.

According to previous studies [23, 41], an example trial of Task 2 is displayed in the bottom panel of Fig. 1. At the onset of each trial, a fixation cross was displayed on the screen for 500 ms. Subsequently, a blank screen was presented for a duration ranging from 800 ms to 1500 ms. Then, trains of three different emotional voices (positive, neutral, and negative voices; presented in a pseudo-random order; each lasting 1000 ms) from the same actor were presented consecutively as a stimulus triplet (S1-S2-S3). S1, S2, and S3 represent the first, second, and third stimuli presented in the triplet, respectively. The inter-stimulus interval ranged from 800 ms to 1500 ms. After the presentation of S1-S2-S3, participants were instructed to respond as accurately and quickly as possible by pressing a key (“1”, “2”, or “3”) to judge whether the last presented stimulus (S3) was positive, neutral, or negative. Key-pressing was counterbalanced across participants to control for potential order effects. Once participants pressed a key or if no response was made within 3 s after the voice presentation, a 200 ms blank screen appeared. Subsequently, two 9-point scales of arousal (1 = *extremely peaceful*, 9 = *extremely excited*) and affective valence (1 = *very unhappy*, 5 = *neutral*, 9 = *very happy*) appeared on the screen. Participants were required to rate their subjective feelings regarding the last presented stimulus (S3) using the keyboard. The inter-trial interval ranged from 1000 ms to 1500 ms. Task 2 comprised a total of 48 trials.

### Statistical analysis

For the analysis of reaction times, arousal ratings, and affective valence ratings, only trials with accurate responses were included (5.64% of the total trials were deleted). Outliers in reaction times exceeding 3 standard deviations from the mean reaction time were removed from each participant’s data (0.72% of the trials were excluded). Reaction times, accuracy, and rating scores of arousal and affective valence were analyzed via a three-way ANOVA with two within-participant factors of “emotion” (positive, neutral, negative) and “stimuli” (S1, S3), as well as the between-participants factor of “group” (High-AQ group, Low-AQ group). If a significant interaction was found, the factor of “group” was further analyzed via post hoc pairwise comparisons. The *p*-values for the main effects and interactions were corrected using the Greenhouse-Geisser method [61]. Statistical significance was set at *p* < 0.05, and post hoc *p*-values were Bonferroni-corrected for multiple comparisons [62–64].

## Results

Mean reaction times, accuracy, and rating scores for arousal and affective valence in response to positive, neutral, and negative voices across all conditions are presented in Table 2. For a comprehensive list of all statistical comparisons, please see Table 3.

For reaction times, a significant main effect of “emotion” was observed (*F*_2, 55_ = 9.90, *p* < 0.001, η^*2*^_p_ = 0.15). The reaction times for neutral voices (1282.30 ms ± 629.38 ms) were shorter than those for positive (1572.22 ms ± 784.34 ms, *p* = 0.001) and negative voices (1521.22 ms ± 696.72 ms, *p* < 0.001). However, there was no significant difference between positive and negative voices (*p* = 0.456). Additionally, a significant main effect of “stimuli” was observed (*F*_1, 56_ = 21.51, *p* < 0.001, η^*2*^_p_ = 0.28). The reaction times for S1 voices (1603.95 ms ± 700.58 ms) were longer than S3 voices (1313.21 ms ± 659.80 ms).

For accuracy, a significant main effect of “group” was found (*F*_1, 56_ = 9.61, *p* = 0.003, η^*2*^_p_ = 0.15). The accuracy of the Low-AQ group (0.97 ± 0.03) was higher than those of the High-AQ group (0.92 ± 0.08). A significant main effect of “emotion” was found (*F*_2, 55_ = 5.68, *p* = 0.004, η^*2*^_p_ = 0.09). The accuracy for neutral voices (0.92 ± 0.12) was lower than those for negative voices (0.97 ± 0.06, *p* = 0.003). However, no significant difference was found between neutral and positive voices (0.95 ± 0.08, *p* = 0.097).

**Fig. 2:**
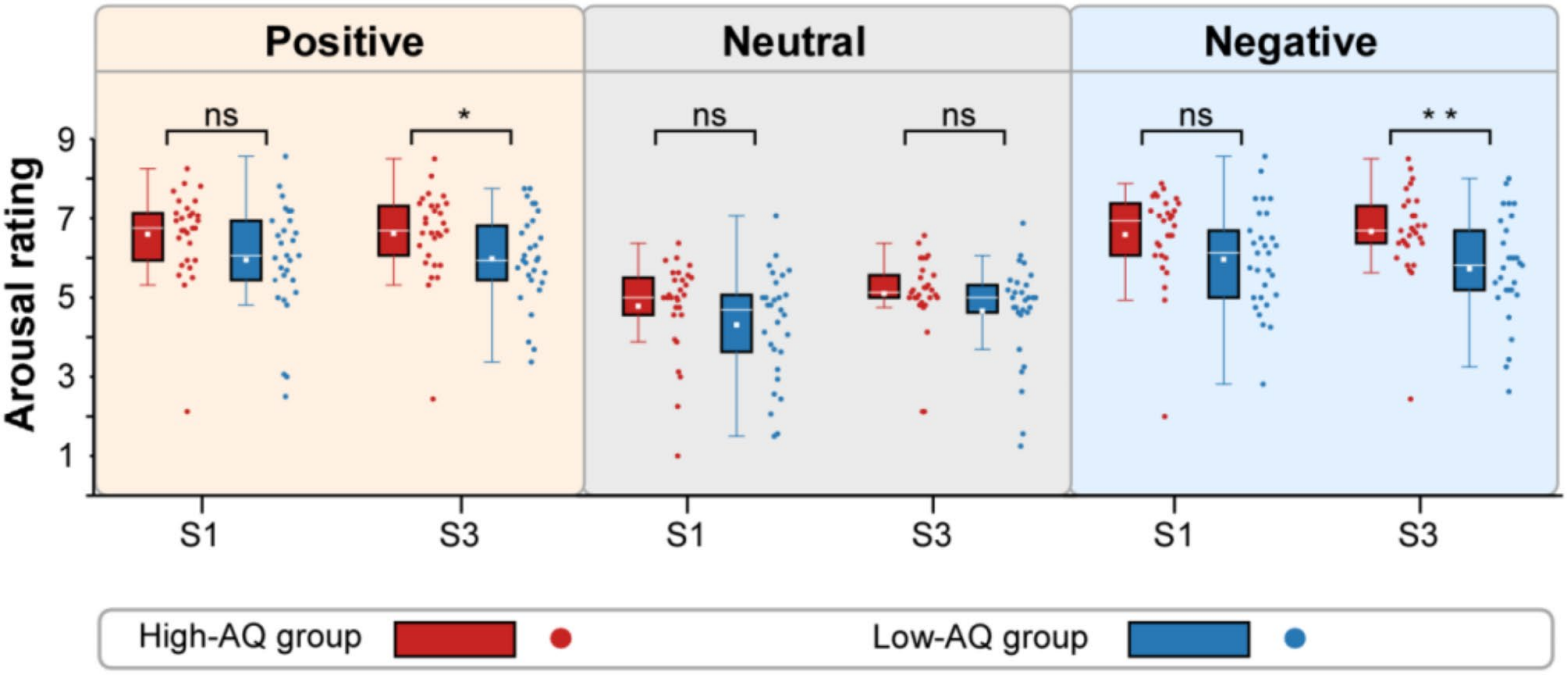
Comparisons of arousal ratings between groups. The arousal ratings of positive, neutral, and negative voices in S1 and S3 were compared between the High-AQ (red) and Low-AQ (blue) groups. Box plots depict the interquartile ranges of the features along with the medians (white lines) and means (white points). ns: *p* > 0.05, *: *p* < 0.05, **: *p* < 0.01

For arousal ratings, a significant main effect of “group” was found (*F*_1, 56_ = 5.38, *p* = 0.024, η^*2*^_p_ = 0.09). The arousal ratings of the High-AQ group (6.06 ± 0.95) were higher than those of the Low-AQ group (5.43 ± 1.10). Additionally, a significant main effect of “emotion” was found (*F*_2, 55_ = 68.45, *p* < 0.001, η^*2*^_p_ = 0.71). The arousal ratings for neutral voices (4.71 ± 1.12) were lower than those for positive (6.29 ± 1.19, *p* < 0.001) and negative voices (6.24 ± 1.25, *p* < 0.001). However, no significant difference was observed between positive and negative voices (*p* = 0.478). A significant interaction of “emotion” x “stimuli” was found (*F*_2, 55_ = 8.54, *p* = 0.001, η^*2*^_p_ = 0.24). As indicated by simple effect analysis, the arousal ratings for neutral voices (S1: 4.55 ± 1.30; S3: 4.88 ± 1.14) were lower than those for positive (S1: 6.27 ± 1.31, *p* < 0.001; S3: 6.30 ± 1.18, *p* < 0.001) and negative voices (S1: 6.28 ± 1.26, *p* < 0.001; S3: 6.20 ± 1.31, *p* < 0.001) in both S1 and S3, but no significant difference was found between positive and negative voices in either the S1 or S3 conditions. A significant interaction of “emotion” x “stimuli” x “group” was found (*F*_2, 55_ = 3.34, *p* = 0.043, η^*2*^_p_ = 0.11). As shown by the simple effect analysis, for S1, no significant difference between groups was found (all *p*s > 0.05). However, for S3, the arousal ratings of the High-AQ group were higher than those of the Low-AQ group to both positive (High-AQ group: 6.62 ± 1.12, Low-AQ group: 5.98 ± 1.16; *F*_1, 56_ = 4.56, *p* = 0.037, η^*2*^_p_ = 0.08) and negative (High-AQ group: 6.67 ± 1.11, Low-AQ group: 5.73 ± 1.35; *F*_1, 56_ = 8.41, *p* = 0.005, η^*2*^_p_ = 0.13) voices, but no group difference was found between the two groups to neutral voices (High-AQ group: 5.10 ± 0.98, Low-AQ group: 4.66 ± 1.26; *F*_1, 56_ = 2.20, *p* = 0.143, η^*2*^_p_ = 0.04). Detailed statistical comparisons for arousal ratings are shown in Fig. 2.

**Table 2:** Results of behavioral data in study 1

Stimuli	Group	Reaction time (ms)				Accuracy				Arousal				Affective valence		
		positive	neutral	negative		positive	neutral	negative		positive	neutral	negative		positive	neutral	negative
S1	High-AQ	1706.95 (876.59)	1551.73 (787.84)	1740.30 (876.37)		0.94 (0.10)	0.85 (0.20)	0.95 (0.08)		6.60 (1.14)	4.78 (1.18)	6.59 (1.17)		6.78 (0.87)	4.65 (0.46)	3.00 (0.81)
	Low-AQ	1642.73 (852.94)	1367.45 (734.68)	1614.56 (794.07)		0.95 (0.07)	0.95 (0.09)	0.98 (0.04)		5.95 (1.40)	4.31 (1.39)	5.97 (1.29)		6.88 (0.99)	4.74 (0.44)	2.94 (1.14)
S3	High-AQ	1486.48 (895.85)	1154.01 (715.98)	1328.42 (657.48)		0.92 (0.11)	0.91 (0.16)	0.96 (0.08)		6.62 (1.12)	5.10 (0.98)	6.67 (1.11)		6.70 (0.97)	4.67 (0.37)	2.93 (0.90)
	Low-AQ	1452.74 (728.26)	1055.99 (606.15)	1401.60 (707.33)		0.97 (0.06)	0.98 (0.06)	0.98 (0.03)		5.98 (1.16)	4.66 (1.26)	5.73 (1.35)		6.68 (1.16)	4.72 (0.45)	3.26 (1.03)

Note: *Mean* (*SD*) of reaction times, accuracy, rating scores of arousal and affective valence in each condition were presented in Table 2

**Table 3:** Summary of statistical analysis of behavioral data in study 1

Variables	Reaction time				Accuracy				Arousal				Affective valence		
	F	*p*	η2 *P*		F	*p*	η2 *P*		F	*p*	η2 *P*		F	*p*	η2 *P*
group	0.18	0.670	0.003		**9.61**	**0.003**	**0.15**		**5.38**	**0.024**	**0.09**		1.57	0.215	0.03
emotion	**9.90**	**< 0.001**	**0.15**		**5.68**	**0.004**	**0.09**		**68.45**	**< 0.001**	**0.71**		**124.87**	**< 0.001**	**0.82**
stimuli	**21.51**	**< 0.001**	**0.28**		2.32	0.133	0.04		1.17	0.284	0.02		0.02	0.881	< 0.001
group × emotion	0.38	0.683	0.01		2.69	0.072	0.05		0.91	0.409	0.03		0.04	0.965	0.001
group × stimuli	0.70	0.405	0.01		0.04	0.853	0.001		0.29	0.594	0.01		0.82	0.370	0.01
emotion × stimuli	1.54	0.219	0.03		2.03	0.142	0.07		**8.54**	**0.001**	**0.24**		2.54	0.088	0.09
group × emotion × stimuli	0.48	0.621	0.01		1.70	0.193	0.06		**3.34**	**0.043**	**0.11**		**3.40**	**0.041**	**0.11**

Note: df: (1, 56). The significant comparisons (*p* < 0.05) were shown in boldface

For affective valence ratings, a significant main effect of “emotion” was found (*F*_2, 55_ = 124.87, *p* < 0.001, η^*2*^_p_ = 0.82). Positive voices (6.76 ± 0.95) were rated as more positive than neutral voices (4.70 ± 0.35, *p* < 0.001) and negative voices (3.03 ± 0.93, *p* < 0.001), and neutral voices were rated as more positive than negative voices (*p* < 0.001). A significant interaction of “emotion” x “stimuli” x “group” was found (*F*_2, 55_ = 3.40, *p* = 0.041, η^*2*^_p_ = 0.11). Simple effects analyses indicated that, for the Low-AQ group, S1 voices were rated as more negative than S3 voices for negative emotions (*F*_1, 56_ = 9.34, *p* = 0.003, η^*2*^_p_ = 0.14, 2.94 ± 1.14 vs. 3.26 ± 1.03), but no significant difference was found in the High-AQ group (*F*_1, 56_ = 0.49, *p* = 0.488, η^*2*^_p_ = 0.01). No significant difference between the groups was found (all *p*s > 0.05).

## Study 2: ERP study

### Method

#### Participants

Six months later, Study 2 re-administered the Mandarin version [57] of the AQ questionnaire [4] to assess autistic traits in 2,325 typically developing students from Chongqing Normal University, China. Consistent with the inclusion criteria and ethical guidelines of Study 1, Study 2 recruited a new sample for the High-AQ and Low-AQ groups from these 2,325 students, thereby avoiding the potential confounding effects of familiarity or habituation. A priori power analysis, performed using G*Power 3, determined that a sample size of 22 participants was necessary to achieve a statistical power of 0.95. This was to detect median-sized effects (f = 0.25) with an alpha value of 0.05 for a three-way ANOVA involving two within-participant factors and one between-participant factor. To mitigate the potential impact of dropouts or errors during the study, Study 2 enrolled 76 participants (High-AQ: *n* = 37, 19 females; Low-AQ group: *n* = 39, 19 females), aged 18–29 years (*Mean* = 21.20 years, *SD* = 2.31 years). Inclusion criteria encompassed normal hearing and the absence of medical, neurological, or psychiatric conditions. No participants were previously diagnosed with ASD. Further details can be found in Table 4.

**Table 4:** Ages and AQ scores of the High-AQ and Low-AQ groups in study 2

Group	Age (years)						AQ score				
	Min	Max	*Mean* ± *SD*	*t*	*p*		Min	Max	*Mean* ± *SD*	*t*	*p*
High-AQ	18	26	21.22 ± 2.16	0.07	0.945		27	35	29.78 ± 1.99	36.60	< 0.001
Low-AQ	18	29	21.18 ± 2.47				7	15	13.10 ± 1.98		

Note: AQ: Autism Spectrum Quotient. The statistical results (*p*-values and *t*-values) were obtained using independent samples *t*-tests between High-AQ and Low-AQ groups

#### Stimuli

Same as study 1.

#### Experimental procedure

The experiment was conducted in a quiet, soundproof room maintained at a comfortable temperature. Stimuli were presented in a pseudo-random order using the E-Prime 3.0 program.

**Fig. 3:**
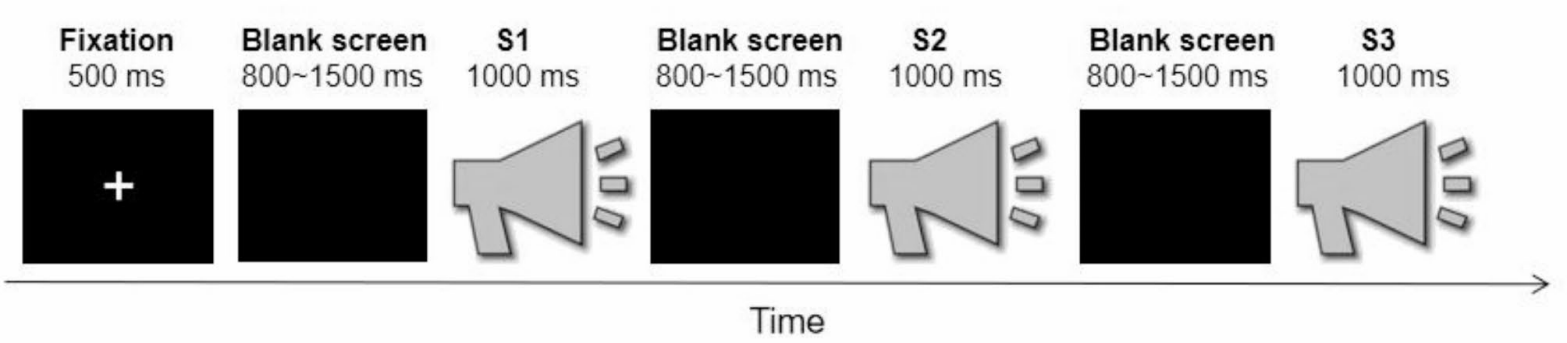
Experimental flowchart for study 2

To avoid attentional interference caused by explicit instructions and to simulate authentic neural responses under naturalistic conditions, participants were instructed to passively listen to the voices while their electroencephalography (EEG) data were recorded. Based on the paradigm of consecutive changeable emotions in the visual modality [23] and the passive listening task [41] used in previous studies on individuals with autistic traits, in each trial of this experiment, three different emotional voices from the same actor were consecutively presented (S1-S2-S3, a triplet). Each trial began with the presentation of a fixation cross on a screen for 500 ms, followed by a blank screen displayed for 800 ms − 1500 ms. Subsequently, S1, S2, and S3 in the triplet were consecutively presented for 1000 ms each, with inter-stimulus intervals ranging from 800 ms to 1500 ms. The inter-trial interval was set at 3 s to 5 s. The experiment comprised two blocks, totaling 240 triplets (80 for each emotional category: positive, neutral, and negative voices). See Fig. 3 for the flowchart of the experiment in Study 2.

#### EEG acquisition and analysis

EEG data were recorded from 64 scalp sites using tin electrodes located according to the international 10–20 system that was mounted on an actiCHamp system (Brain Vision LLC, Morrisville, NC, USA). EEG activities were amplified with a 0.01 Hz − 100 Hz bandpass and were continuously sampled at 1000 Hz. The electrode at the bilateral mastoids was used as the recording reference, and the electrode on the medial frontal aspect was used as the ground electrode. All electrode impedances remained below 5 kΩ.

In this study, EEG data were pre-processed and analyzed using MATLAB R2016a (MathWorks, USA) and the EEGLAB toolbox [65]. Continuous EEG signals were band-pass filtered 0.1 Hz − 40 Hz. Time windows spanning 200 ms before and 1,000 ms after the onset of stimuli were extracted from the continuous EEG data. The extracted windows were then baseline-corrected using the 200 ms time interval prior to stimulus onset. Moreover, epochs with amplitude values exceeding ± 80 µV at any electrode were excluded from further analyses. Epochs were visually inspected for trials contaminated by gross movements, and those trials were also excluded. Eye movement artifacts were corrected using the independent component analysis (ICA) algorithm [66]. Four participants (one participant from the High-AQ group and three participants from the Low-AQ group) were excluded due to excessive artifacts in the EEG recordings. Finally, data from the High-AQ group (*n* = 36, 19 females) and the Low-AQ group (*n* = 36, 16 females) were included in the analysis. The removed EEG epochs constituted 3.39% ± 5.51% of the total number of trials. The EEG epochs elicited by positive, neutral, and negative voices were averaged, and the times were locked to the beginning of the stimuli, yielding nine averaged waveforms.

According to the topographical distributions of the grand-averaged ERP activity and previous studies [41, 50], the dominant ERP components analyzed in this study were identified as N1, P2, and LNC. Specifically, both N1 and P2 were assessed at the electrode sites FC1, FCZ, FC2, C1, CZ, and C2, and the latency intervals were 145 ms − 175 ms and 225 ms − 255 ms, respectively. The LNC was measured at the electrode sites F1, FZ, F2, FC1, FCZ, and FC2, and the latency interval was 400 ms − 600 ms.

### Statistical analysis

ERP data were analyzed via a three-way repeated-measures ANOVA, with two within-participant factors of “emotion” (positive, neutral, negative) and “stimuli” (S1, S2, S3), as well as the between-participants factor of “group” (High-AQ group, Low-AQ group). If a significant interaction was found, the factor of “group” was further analyzed via post hoc pairwise comparisons. The degrees of freedom for *F*-ratios were corrected using the Greenhouse-Geisser method [61]. Statistical significance was set at *p* < 0.05, and post hoc *p*-values were Bonferroni-corrected for multiple comparisons [62–64].

## Results

**Fig. 4:**
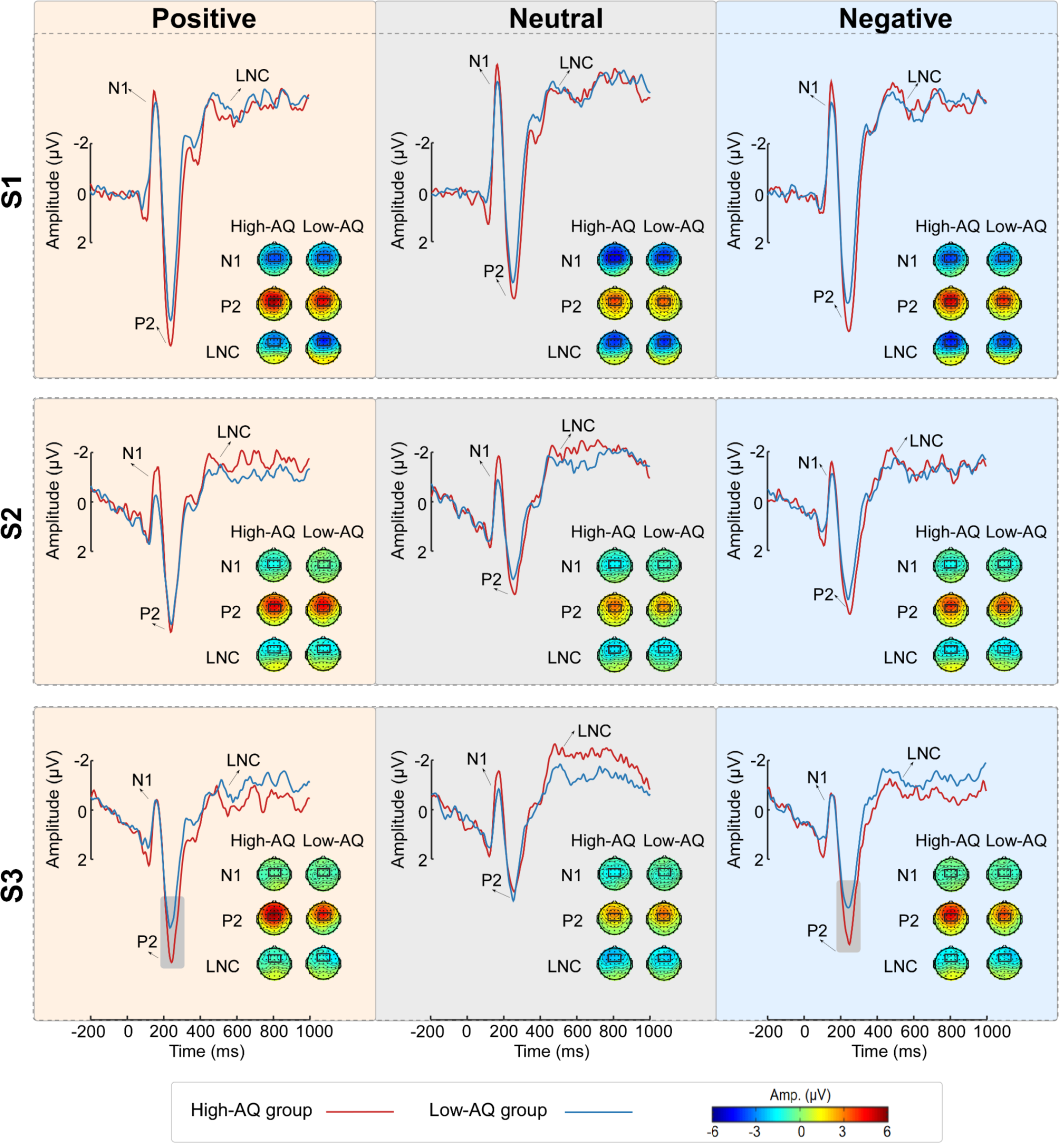
ERP responses in Study 2. ERP waveforms of the High-AQ (red lines) and Low-AQ (blue lines) groups to positive (orange background), neutral (gray background), and negative voices (blue background). S1 (top panel), S2 (middle panel), and S3 (bottom panel) represent the first, second, and third voices presented in the S1-S2-S3, respectively. Scalp topographies of the dominant ERP components (N1, P2, and LNC) were computed at respective peak latencies. Electrodes estimating ERP amplitudes were marked using black squares on the corresponding scalp topographies

**Fig. 5:**
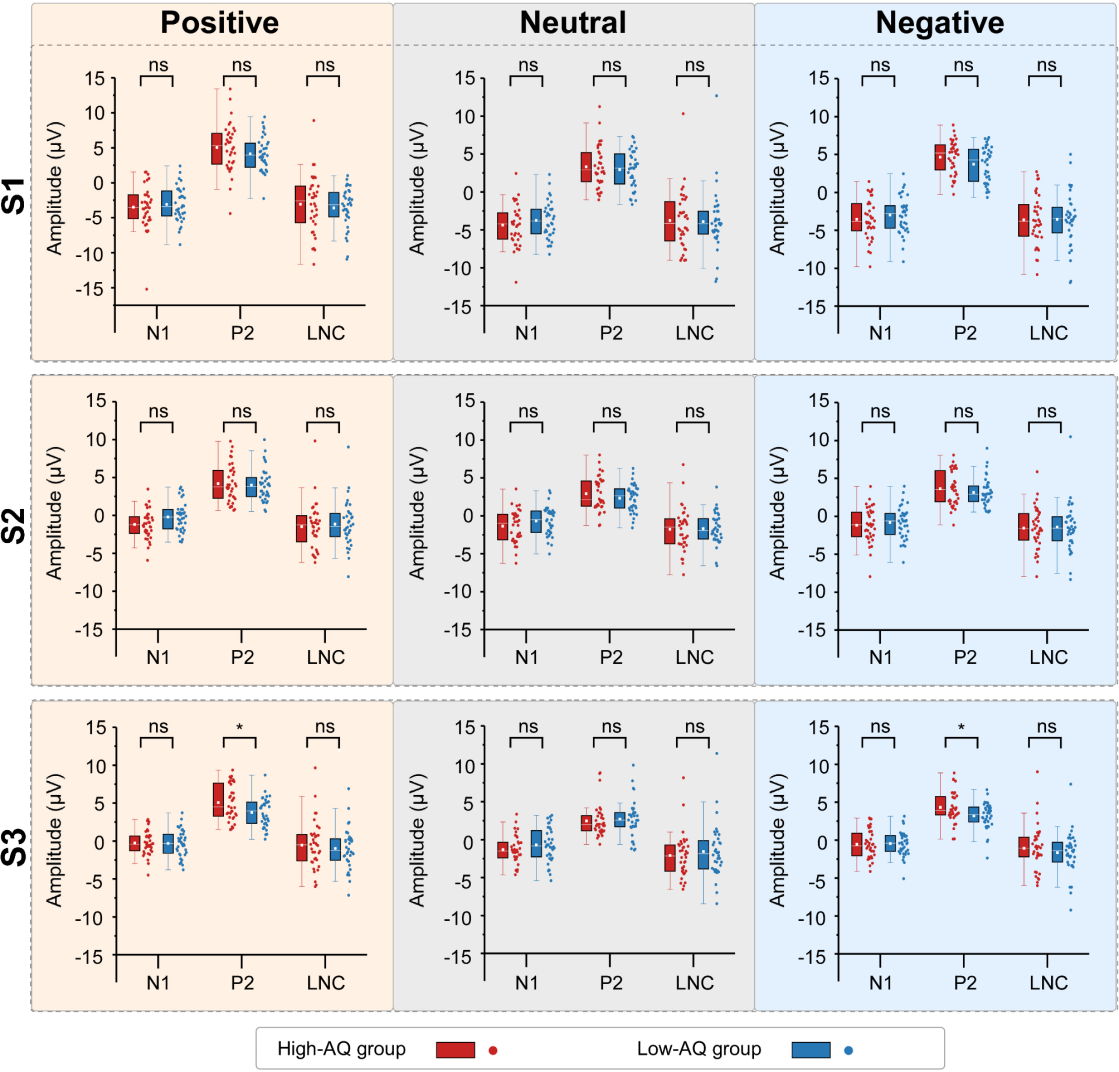
Comparisons of ERP amplitudes between groups. The amplitudes of positive (orange background), neutral (gray background), and negative voices (blue background) for S1 (top panel), S2 (middle panel), and S3 (bottom panel) were compared between the High-AQ (red) and Low-AQ (blue) groups. Box plots illustrate the interquartile ranges of the features along with the medians (white lines) and means (white points). ns: *p* > 0.05, *: *p* < 0.05

Averaged ERP waveforms and scalp topographies of the dominant ERP components in response to emotional voices are shown in Fig. 4. Detailed statistical comparisons for ERP amplitudes can be found in Table 5; Fig. 5.

**N1** The N1 amplitudes were influenced by the main effects of “emotion” (*F*_2, 69_ = 13.65, *p* < 0.001, η^*2*^_p_ = 0.16) and “stimuli” (*F*_2, 69_ = 117.19, *p* < 0.001, η^*2*^_p_ = 0.77). Neutral voices (-2.03 µV ± 1.92 µV) were more negative than both positive (-1.43 µV ± 1.93 µV, *p* < 0.001) and negative (-1.59 µV ± 1.90 µV, *p* < 0.001) voices. However, no significant difference was observed between positive and negative voices (*p* = 0.137). S1 voices (-3.54 µV ± 2.44 µV) were more negative than both S2 (-0.91 µV ± 1.85 µV, *p* < 0.001) and S3 (-0.60 µV ± 1.61 µV, *p* < 0.001) voices, and S2 voices were more negative than S3 voices (*p* = 0.004).

**Table 5:** Summary of statistical analysis of ERP amplitudes

Variables	N1				P2				LNC		
	F	*p*	η2 *P*		F	*p*	η2 *P*		F	*p*	η2 *P*
group	1.23	0.271	0.02		2.20	0.143	0.03		0.01	0.931	< 0.001
emotion	**13.65**	**< 0.001**	**0.16**		**36.44**	**< 0.001**	**0.51**		**8.23**	**0.001**	**0.19**
stimuli	**117.19**	**< 0.001**	**0.77**		**5.79**	**0.005**	**0.14**		**64.90**	**< 0.001**	**0.65**
group × emotion	0.77	0.464	0.01		1.78	0.177	0.05		0.57	0.571	0.02
group × stimuli	2.50	0.090	0.07		0.89	0.417	0.03		1.13	0.329	0.03
emotion × stimuli	1.91	0.119	0.10		0.79	0.535	0.01		1.92	0.117	0.10
group × emotion × stimuli	1.96	0.111	0.11		**2.62**	**0.035**	**0.04**		1.75	0.149	0.10

Note: df = (1, 70). The significant comparisons (*p* < 0.05) were shown in boldface

**P2** The P2 amplitudes were influenced by the main effects of “emotion” (*F*_2, 69_ = 36.44, *p* < 0.001, η^*2*^_p_ = 0.51) and “stimuli” (*F*_2, 69_ = 5.79, *p* = 0.005, η^*2*^_p_ = 0.14). Neutral voices (2.78 µV ± 2.03 µV) were smaller than positive (4.37 µV ± 2.17 µV, *p* < 0.001) and negative voices (3.78 µV ± 1.77 µV, *p* < 0.001), and positive voices were larger than negative voices (*p* < 0.001). S1 voices (3.96 µV ± 2.31 µV) were larger than S2 voices (3.37 µV ± 1.88 µV, *p* = 0.002). No significant difference was found between S2 and S3 voices (3.59 µV ± 1.81 µV, *p* = 0.068). The P2 amplitudes were influenced by the interaction of “group” × “emotion” × “stimuli” (*F*_4, 67_ = 2.62, *p* = 0.035, η^*2*^_p_ = 0.04). Simple effects analyses indicated that, for positive and negative voices, the P2 amplitudes of the High-AQ group were larger than those of Low-AQ group to S3 voices (positive: 5.06 µV ± 2.34 µV vs. 3.78 µV ± 1.88 µV, *F*_1, 70_ = 6.54, *p* = 0.013, η^*2*^_p_ = 0.09; negative: 4.32 ± 2.04 µV vs. 3.20 µV ± 1.75 µV, *F*_1, 70_ = 6.28, *p* = 0.015, η^*2*^_p_ = 0.08). However, no significant difference between groups was found for neutral voices or other stimuli (S1 and S2 voices) (all *p*s > 0.05).

**LNC** The LNC amplitudes were influenced by the main effects of “emotion” (*F*_2, 69_ = 8.23, *p* = 0.001, η^*2*^_p_ = 0.19) and “stimuli” (*F*_2, 69_ = 64.90, *p* < 0.001, η^*2*^_p_ = 0.65). Positive voices (-1.80 µV ± 2.93 µV) were more positive than both neutral (-2.47 µV ± 2.95 µV, *p* < 0.001) and negative voices (-2.15 µV ± 2.83 µV, *p* = 0.017). However, no significant difference was found between neutral and negative voices (*p* = 0.086). In addition, S1 voices (-3.59 µV ± 3.38 µV) exhibited larger amplitudes than S2 (-1.52 µV ± 2.58 µV, *p* < 0.001) and S3 voices (-1.32 µV ± 2.76 µV, *p* < 0.001). No significant difference was found between S2 and S3 voices (*p* = 0.146).

## Discussion

The primary aim of this research was to investigate the behavioral and neural responses of individuals with autistic traits to consecutive changeable emotions in the auditory modality. The results indicated that for the consecutive changeable voices (S3), the High-AQ group exhibited higher arousal ratings and larger P2 amplitudes for both positive and negative voices compared to the Low-AQ group. However, for the single/first voices (S1), no significant group difference was observed. These findings indicate that individuals with autistic traits may exhibit heightened arousal and altered neural processing in response to consecutive changeable emotions in the auditory modality.

Main effects of “emotion” were found in both Study 1 and Study 2. In line with previous studies [58, 67], Study 1 showed that negative and positive voices were associated with longer reaction times and higher arousal ratings than neutral voices. In addition, positive voices were rated more positively than both neutral voices and negative voices, and neutral voices were rated more positively than negative voices. Consistent with previous ERP research [68], Study 2 demonstrated that positive and negative stimuli elicited larger P2 amplitudes than neutral stimuli. As the P2 component is known to be sensitive to the arousal levels of auditory stimuli [53–55], these main effects of “emotion” in both behavioral and ERP responses suggest that positive and negative voices induced higher mental arousal than neutral voices.

Additionally, aligning with previous research [69–72], the present study also found that the main effects of “group” influenced both accuracy and arousal. Lower accuracy and higher arousal ratings were found in the High-AQ group than the Low-AQ group, suggesting that individuals with autistic traits may exhibit challenges in recognizing emotional voices and experience higher arousal levels in response to emotional voices.

In line with our previous study in the visual modality [23], significant interactions of “group” × “emotion” × “stimuli” were found in both Study 1 and Study 2 of the present auditory research. Relative to the Low-AQ group, the High-AQ group showed higher arousal ratings and larger P2 amplitudes to S3 voices for both positive and negative voices, but they exhibited similar behavioral and neural responses to S1 voices. Given that P2 amplitudes are known to be sensitive to the arousal levels of emotional stimuli [53–55], these results suggest that, compared to the Low-AQ group, the High-AQ group may exhibit enhanced emotional arousal to S3 voices but similar behavioral and neural responses to S1 voices. S1 voices in both Study 1 and Study 2 were the single/first presented stimuli and were not disturbed by the preceding stimuli, whereas S3 voices were the last presented stimuli in the trains of three consecutive emotional voices (S1-S2-S3) and could be disturbed by the previously presented S1 and S2 voices. Thus, these results suggest that individuals with autistic traits may exhibit enhanced emotional arousal in both behavioral and neural responses to consecutive changeable emotional stimuli rather than single emotional stimuli in the auditory modality.

The aforementioned results suggest that individuals with autistic traits exhibit heightened behavioral and neural responses only to consecutive changeable emotional voices, with no such response to single emotional stimuli. This may be attributed to the complexity and intensity of consecutive changeable emotional voices, which are influenced by preceding stimuli and pose greater challenges than single emotional stimuli. Additionally, heightened arousal ratings and ERP responses were observed only in response to more intense positive and negative emotions rather than low-intensity neutral voices. This pattern may reflect an exaggerated emotional response to a sequence of highly intense emotional stimuli in individuals with autistic traits, as explained by the intense world theory [73, 74]. This theory posits that individuals with ASD perceive sequences of emotional cues as overwhelmingly intense, leading to heightened attention, processing, memory, and emotional reactivity to these stimuli. Such heightened responses may induce sensory fragmentation and overload, resulting in overwhelming stress and aversion, potentially leading to social avoidance behaviors, such as avoiding eye contact, withdrawing from interactions, and limiting communication. Consequently, the social challenges observed in individuals with autistic traits or ASD may not stem from insufficient processing of emotional stimuli, but rather from excessive processing and heightened responses to intense, consecutive, and changeable emotional stimuli [73, 74].

An alternative explanation for the heightened behavioral and neural responses to consecutive changeable emotional voices in individuals with autistic traits may involve challenges in mental flexibility. Mental flexibility, also known as task switching, refers to the ability to shift attention from one rule, feature, or task to another during task execution [75, 76], which is crucial for adapting to changing demands, managing complex environments efficiently, and handling multitasking scenarios [77]. Previous studies have shown that, compared to control groups, individuals with ASD exhibit greater switching costs when processing complex, unpredictable (i.e., variable), and implicit (i.e., not guided by explicit rules) social-emotional transition tasks (emotional flexibility tasks) [78]. Further research on emotional flexibility in social versus non-social contexts found that individuals with ASD display higher switching costs (longer reaction times and lower accuracy) only in social contexts, with no significant differences from non-autistic individuals in non-social contexts [79]. These findings suggest that individuals with ASD require significantly more cognitive resources than typically developing individuals to adapt to complex, variable social-emotional contexts, as opposed to non-social emotional environments. Additionally, an ERP study has shown that, compared to the Low-AQ group, the High-AQ group exhibits greater switching costs and more pronounced ERP responses during emotional task-switching, indicating higher demands for conflict inhibition and greater cognitive resource allocation in managing emotional task-switching [80], which may facilitate monitoring and adaptation to changes [81]. In the present study, the S1-S2-S3 paradigm involved the consecutive presentation of three different social-emotional voices. This design required participants to process consecutive, changeable social-emotional stimuli, rather than the single social-emotional stimuli commonly used in previous research [42, 82], thereby necessitating adaptation to continuously shifting task demands, incurring higher switching costs, and demanding greater mental flexibility. This may explain the heightened behavioral and neural responses observed in individuals with autistic traits, as they mobilize additional cognitive resources to adapt to emotional variability.

In fact, these insights not only deepen theoretical understanding but also advance ASD research at both methodological and applied levels. Theoretically, this study aligns with previous investigations in the visual modality [23], further revealing that individuals with autistic traits or ASD exhibit heightened emotional arousal and neural responses when processing consecutive changeable emotional voices in the auditory modality, whereas no significant differences are observed in response to single emotional voices. This finding underscores the importance of auditory cues in emotional processing, enriches the overall comprehension of multimodal emotion processing, and provides crucial evidence supporting the intense world theory and the notion of reduced mental flexibility in ASD individuals. It provides a novel theoretical perspective to explain the challenges faced by individuals with ASD in processing dynamically changing emotional information in real social environments. Methodologically, the S1-S2-S3 paradigm simulates the complexity of dynamically changing emotional stimuli encountered in daily life, offering higher ecological validity and a closer approximation to real-world social scenarios compared to traditional single static stimulus experiments. Additionally, by integrating psychology and neuroscience through cross-dimensional and interdisciplinary analyses of behavioral and neuroimaging data [83], this study comprehensively delineates the unique patterns of emotion processing in individuals with ASD and provides a reference for optimizing experimental design. From an applied perspective, this study further validates that individuals with ASD struggle to effectively regulate their emotions to cope with continuously changing emotional environments [84]. Consequently, future interventions should prioritize enhancing emotion regulation capabilities and mental flexibility in individuals with ASD. Furthermore, intervention environments should minimize the intensity and variability of social-emotional cues as much as possible, creating quiet, predictable, and low-stimulus variation social contexts to reduce the interference of sensory overload on emotional processing [85]. This empirical intervention approach not only aids in improving the social performance of individuals with ASD but also establishes a robust theoretical foundation for precise interventions and personalized treatments.

Despite the potential implications, several limitations of the current study should be noted. First, considering the increased likelihood of false positives when conducting multiple analyses across different ERP components [63], future research should increase the sample size to both replicate and demonstrate the robustness of our findings. Second, studies on autistic traits in the general population cannot fully substitute for studies involving clinically diagnosed ASD individuals [86]. Future research should include participants with clinically diagnosed ASD to verify whether the effects observed in individuals with autistic traits generalize to the ASD population. Third, the present study utilized an extreme groups approach (top and bottom 10% of AQ scores), which may limit the generalizability of the findings. Additionally, having participants complete a self-report AQ questionnaire and indicate whether they have ever been diagnosed with ASD may introduce bias and inaccuracies due to inaccurate self-assessment or social desirability effects. Future research should incorporate a more representative and diverse sample. Inclusion criteria should be further validated through clinical assessments and corroborated by parent or teacher reports. Fourth, the “double empathy problem” [87, 88] suggests that individuals with ASD experience challenges in understanding and processing the emotional expressions of typically developing individuals. Conversely, typically developing individuals also face difficulties in comprehending the emotions and thoughts of individuals with ASD. Future research should further investigate how these bidirectional challenges affect social interactions and explore potential interventions to enhance mutual understanding between individuals with ASD and typically developing individuals. Fifth, previous studies have found that individuals with ASD exhibit difficulties in emotional flexibility specifically with social stimuli, but not with non-social stimuli [79]. Therefore, future research could further investigate differences in arousal responses to consecutive changeable social and non-social emotional stimuli in individuals with autistic traits or ASD to determine whether their challenges with mental flexibility are primarily associated with social contexts. Finally, individuals with autistic traits or ASD typically exhibit higher levels of anxiety and social anxiety compared to control groups [89, 90], both of which have been shown to impact emotional processing [91, 92]. Thus, future research should focus on distinguishing the unique contributions of autistic traits to emotional processing from other potential confounding factors, such as social anxiety.

Moreover, traditional psychiatric models, which often focus on single factors or perspectives [93], inadequately address the complex underlying mechanisms of ASD, thereby catalyzing the emergence of interdisciplinary integrative research approaches [94]. In this context, Luo et al. [83] introduced the concept of “culturomics,” which advocates a cross-level, interdisciplinary, and systematic research framework. This framework integrates multidimensional data across micro, meso, and macro levels by incorporating advanced analytical tools such as deep neural networks (DNN) and representational similarity analysis (RSA), thereby uncovering the deep-seated mechanisms and patterns underlying cultural phenomena. Building on this foundation, future research on emotion recognition in individuals with ASD can advance in several promising directions. First, integrating multiple disciplines such as psychology, neuroscience, and genetics to construct a cross-level, cross-dimensional and interdisciplinary research model that combines genetic factors, biological neural networks, and behavioral manifestations will systematically reveal the mechanisms of emotion recognition in ASD. Second, utilizing technologies such as DNN and RSA to analyze high-dimensional data across modalities and scales [95] will enable the extraction of neural encoding patterns of emotional information in individuals with ASD. This approach will facilitate the construction of multimodal emotion models, promote cross-cultural and cross-individual studies, and optimize personalized intervention strategies. Third, the application of artificial intelligence (AI) technologies in ASD intervention and screening has demonstrated significant efficacy [96, 97]. Future research should integrate psychological and neuroscientific data to develop AI-assisted diagnostic and intervention tools using deep learning and machine learning techniques, thereby enhancing diagnostic accuracy and efficiency [98] and facilitating personalized treatment strategies [99].

## Conclusions

The aim of this research was to investigate the behavioral and neural responses of individuals with autistic traits to consecutive changeable emotions in the auditory modality. Individuals with autistic traits exhibited higher arousal levels to consecutive changeable emotional voices, but no group differences were found in response to single emotional voices. These insights not only enhance our theoretical understanding but also offer potential practical approaches to support individuals with autistic traits and ASD in managing emotions in their daily lives.

## Data Availability

The datasets used and/or analyzed during the current study are available from the corresponding author on reasonable request.

## Abbreviations

ASD: Autism spectrum disorder
AQ: Autism-spectrum quotient.
